# Bridging Implementation, Knowledge, and Ambition Gaps to Eliminate Tuberculosis in the United States and Globally

**DOI:** 10.3201/eid1703.110031

**Published:** 2011-03

**Authors:** Kenneth G. Castro, Philip LoBue

**Affiliations:** Author affiliation: Centers for Disease Control and Prevention, Atlanta, Georgia, USA

**Keywords:** Bacteria, tuberculosis and other mycobacteria, global health, perspective

## Abstract

We reflect on remarkable accomplishments in global tuberculosis (TB) control and identify persistent obstacles to the successful elimination of TB from the United States and globally. One hundred and twenty nine years after Koch’s discovery of the etiologic agent of TB, this health scourge continues to account for 9.4 million cases and 1.7 million deaths annually worldwide. Implementation of the Directly Observed Treatment Short-course strategy from 1995 through 2009 has saved 6 million lives. TB control is increasingly being achieved in countries with high-income economies, yet TB continues to plague persons living in countries with low-income and lower-middle–income economies. To accelerate progress against the global effects of disease caused by TB and achieve its elimination, we must bridge 3 key gaps in implementation, knowledge, and ambition.

As we commemorate World Tuberculosis (TB) Day, March 24, we pause to reflect on remarkable accomplishments in eliminating TB in the United States and other parts of the world and to identify persistent obstacles to its eventual elimination. World TB Day marks the day when, in 1882, Robert Koch delivered his lecture to the Physiologic Society of Berlin announcing the discovery of the tubercle bacillus as the etiologic agent of TB (*1*). At the time, TB was estimated to account for one fifth to one fourth of all deaths in Europe. One hundred twenty-nine years later, TB is increasingly under control in most countries with high-income economies (*2*) yet continues to afflict persons living in countries with low-income and lower-middle–income economies (*3**,**4*). The World Health Organization (WHO) reported an estimated 9.4 million incident TB cases and 1.7 million deaths in 2009. Existing evidence-based interventions for TB control that have been successfully implemented from 1995 through 2009 have saved 6 million lives and alleviated much human suffering (*3*). Yet, by 2009 only an estimated 63% of annual incident TB cases were being detected and reported; of these, 86% were successfully treated (*3*). To accelerate progress against the global effect of disease caused by TB and to achieve its elimination, we must bridge 3 key gaps in implementation, knowledge, and ambition.

## Implementation Gap

In his 1963 lecture delivered at the Postgraduate Medical School in London, Wallace Fox observed that remarkable progress in the chemotherapy of TB had been achieved over the prior decade “in the technically advanced countries” (*5*). In contrast, he remarked that nonindustrialized countries “have derived very little benefit from the progress.” Fox cited 2 reasons for this lack of progress: a shortage of medical resources and “little attempt to adapt present knowledge to their specific problems” (*5*). During Fendall’s 1972 presentation at the Symposium on the Teaching of Teaching Tropical Medicine, an epitaph is suggested to describe medicine throughout the 20th century: “Brilliant in its scientific discoveries, superb in its technological breakthroughs, but woefully inept in its application of knowledge to those most in need” (*6*). Fendall further suggested “all that remains is the problem of translating what is current common knowledge and routine medical and health practice to the other two thirds of the world: the ‘implementation gap’ must be closed.”

Globally, this implementation gap has been closing as a result of reliance on the evidence-based strategy for TB control, originally known as Directly Observed Treatment Short-course (DOTS). This strategy was initially based on diagnostic and treatment recommendations derived from randomized controlled trials, conducted largely by the British Medical Research Council (*7*) and the US Public Health Service, which established the efficacy and safety of drugs against TB (*8*). Additionally, the basic elements of the strategy were defined and field tested under mutual assistance programs between host countries and the International Union Against Tuberculosis and Lung Disease (*9*). The DOTS strategy was endorsed by consensus derived in technical advisory bodies and promulgated by WHO and the global Stop TB Partnership. Its widespread implementation has been more recently facilitated by resources from governments; the Global Fund for AIDS, Tuberculosis, and Malaria; and the President’s Emergency Plan for AIDS Relief (*10**–**12*). Furthermore, TB control has been demonstrated to be among the most cost effective of health interventions (*13*).

The original DOTS strategy contained 5 basic elements: 1) secure political commitment with adequate and sustained financing; 2) ensure early case detection and diagnosis through quality-assured bacteriology; 3) provide standardized treatment with supervision and patient support; 4) ensure effective drug supply and management; and 5) monitor and evaluate performance and effects. This strategy has now been expanded to contain additional elements to confront other evolving needs, such as addressing HIV-associated TB, and multidrug-resistant and extensively drug-resistant TB; contributing to strengthening health systems; engaging all providers (public, voluntary, and private) and affected communities; promoting use of the International Standards for TB Care; and enabling and promoting research (*14**,**15*).

The advances achieved with DOTS from 1995 through 2009 include treating nearly 49 million persons and curing 41 million with TB, which was accompanied by a peak in global TB trends in 2004 followed by a relatively slow decline (*3*). These advances notwithstanding, TB continues to hold its dubious place as a leading infectious killer of young adults, and the disease preys on the most vulnerable persons in many parts of the globe (*16*). These populations are known to have difficulty in accessing available diagnostic tests and in obtaining curative short-course therapeutic regimens that require >6 months of multiple drugs to achieve the desired outcomes. Even when all countries of the world have adopted policies consistent with the DOTS strategy, a sizable proportion of estimated cases (≈37%) are undetected, and those infected are likely not receiving optimal treatment regimens. Efforts must now focus on tackling social determinants of illness associated with TB by expanding and facilitating access to impoverished persons in densely populated urban areas and remote villages.

In addition to partnering with all health providers (e.g., private, public, voluntary, traditional healers) to facilitate access to care, those concerned with public health must concentrate on subtleties such as optimizing the number of clinics or dispensaries offering diagnostic and therapeutic services, providing patient-convenient hours of operation, recognizing difficulties with distance and transportation, and minimizing out-of-pocket expenditures (including lost wages) for transportation, child care, and diagnostic services. An analysis from India has reported that 72% of TB patients who had a low standard of living (e.g., earning US $1–$2/d) first saw private providers and spent, on average, $145 before starting treatment with the Revised National TB Program, thus documenting the devastating economic toll incurred by poor persons with TB (*17*). Engagement of affected communities will also prove crucial to create educated consumers of services. Public communication campaigns will help educate persons about the signs and symptoms of TB, provide information about where to access quality services and drugs, alleviate stigma, and create the demand for these basic health services from all providers of care and government decision-makers.

Other scientific advances that have lagged behind in implementation include the use of universal genotyping of *Mycobacterium tuberculosis* clinical isolates as a way to understand and interrupt chains of recent and ongoing transmission and the use of universal drug susceptibility testing with liquid culture media that reduce turn-around times by several weeks (available in the United States since 1994) for timely surveillance of drug resistance trends and to guide optimal treatment regimens. Most recently, technologic advances have demonstrated the ability to rely on detection of bacterial DNA by PCR. The WHO policy recommendation to rely on Xpert MTB/RIF (Cepheid, Sunnyvale, CA, USA) for same-day diagnosis accentuates and magnifies this implementation gap (*18*).

A growing concern has to do with the gap in successfully addressing concurrent conditions associated with TB, such as HIV, diabetes, smoking, indoor air pollution, alcoholism, and malnutrition (*16*). This more holistic approach provides an ideal way to benefit both individual and public health, and secondarily to strengthen health systems. When modeled after the basic principles that underpin TB control, the combined interventions will provide platforms for planning, service delivery, analysis, accountability, and corrective actions.

In the zeal to bridge the implementation gap, we must avoid past false dichotomies. There are those who see the way forward as limited to securing investments and channeling all resources to expand access to available diagnostic services and curative drugs. Available tools are relatively blunt and limited, especially for effectively addressing HIV-associated TB and multidrug-resistant and extensively drug-resistant TB. In tackling urgent unmet needs, we must honestly acknowledge existing limitations and not ignore the need to bridge the immense knowledge gap in TB. Otherwise, we risk interventions that lack innovation, creativity, and do not keep pace with technological advances that could accelerate the path to elimination.

## Knowledge Gap

There remain critical areas of collective ignorance with regard to *M. tuberculosis.* These include knowledge of rapid, simple, and inexpensive methods of detection; molecular mechanisms of resistance to chemotherapy; virulence; host defense correlates of susceptibility to and protection against the organism; and optimal targets for development of new antimicrobial drugs.

Until the past 2 decades, definitive detection of *M. tuberculosis* relied exclusively on culture, which takes weeks because of the requisite generation time of 18–24 hours, giving rise to the apt descriptor of *M. tuberculosis* as “slow growing” bacteria. In low resource settings, even culture may not be available and diagnosis must be based on smear microscopy, which fails to detect nearly half of patients with TB (*14*). Advances in molecular biology and, ultimately, the sequencing of the *M. tuberculosis* genome led to rapid molecular methods of detection that, although reasonably accurate, were cumbersome and expensive (*19**,**20*). Only relatively recently has a promising new molecular diagnostic test become available, the Xpert TB/RIF, that is both simple and accurate (*21*). However, even with concessionary pricing for low-income countries, cost still remains an issue.

Similarly, detection of drug resistance almost solely relies on phenotypic culture-based methods. Here, also, advances in molecular biology are moving the field forward, but the situation is more complex than for detection of the organism. Fortunately, for the most important anti-TB drug, rifampin, >95% of resistance can be attributed to mutations in 1 gene, which has greatly simplified the development of molecular tests to detect rifampin resistance (*22*). Commercial assays that use line-probe and molecular beacon technologies have been produced that are rapid and accurate (*22**,**23*). However, for other first-line drugs (such as isoniazid and ethambutol) and second-line drugs, the molecular mechanisms of drug resistance have only been partially elucidated, inhibiting the development of rapid molecular assays for these drugs (*24*). Thus, there remains a heavy reliance on inefficient and slow culture-based phenotypic methods.

Virulence of *M. tuberculosis*, especially variation among strains, is also poorly understood. There is evidence suggesting some strains may result in higher rates of disease progression, treatment failure, and relapse (*25*). Identifying genetic markers of *M. tuberculosis* virulence would enable additional attention to be focused on patients infected with strains manifesting such markers and who are therefore at the greatest risk for poor outcomes.

Our lack of understanding of host defense correlates of susceptibility to and protection against *M. tuberculosis* has stymied progress in 2 key areas: vaccine development and prevention through treatment of latent TB. A vaccine that uses an attenuated strain of *M.* *bovis* (*M. bovis* BCG) has been available for nearly a century and is one of the most widely used vaccines in the world. Although the vaccine does offer substantial protection against dissemination of *M. tuberculosis* infection in children, it only provides modest and highly variable protection against TB in general (*26**,**27*). Clearly, more efficacious and safe vaccines are needed; these are only likely to be produced through a better understanding of immunologic mechanisms and correlates of protection. A related knowledge gap is the lack of understanding of why only a small fraction (≈5%–10%) of persons infected with *M. tuberculosis* later exhibit disease (*28*). It is evident that immunocompromised persons (e.g., HIV infected or receiving tumor necrosis–α inhibitors) are at greater risk, but we have little knowledge of why certain persons with apparently healthy immune systems experience progression to illness (*28*). This results in treating 10–20 persons with latent TB for every 1 that will have the infection progress to disease. Given the length of optimal treatment (9 months) and potential toxicity (liver injury), this intervention is obviously suboptimal and could be made much more efficient if it could be targeted to persons at the highest risk of becoming ill. Thus, there is a crucial need to find genetic and immunologic markers that confer increased susceptibility to progression.

Standard TB treatment requires multiple drugs for >6 months’ duration (*29*). These drugs have multiple and overlapping toxicities. For drug-resistant TB, treatment consists of more toxic, less effective second-line drugs that must be taken for 18–24 months (*29*). Some patients with extensively drug-resistant TB have been described as having run out of realistic therapeutic options and thus resemble TB patients in the pre–antimicrobial drug era. Additionally, persons with latent TB who are not ill tend to have a difficult time completing the 9 months required for isoniazid treatment (previously described as preventive therapy or chemoprophylaxis). Safe and effective regimens that could be administered intermittently and/or within 3 months are under study and show promise (*30*). All these factors underscore the need for new medications that are better tolerated and can produce a cure in less time. Given that drug toxicity and resistance are often class effects, development of new classes of anti-TB drugs is another essential research need. Such development, in turn, will likely require identification of new and better drug targets.

Although the knowledge and implementation gaps must be bridged, simply rectifying these inadequacies is insufficient for elimination of TB. Societal resolve and ambition are also required to garner the necessary resources for sustained efforts and effective programs, adapted to local epidemiologic realities.

## Ambition Gap

The report from the 1959 Arden House Conference on TB made a daring statement, possibly well ahead of its time, indicating that TB control “has progressed to the point where virtual elimination of the disease as a public health problem appears to be within reach” (*31*). However, it was not until 3 decades later that the Centers for Disease Control published a formal consensus plan for the elimination of TB in the United States (*32*). This plan was ambitious, yet initially naive about the full extent of the effects on TB incidence due to HIV infection; multidrug resistance; institutional transmission of *M. tuberculosis*; and the time lag for the development of new technologies for more effective prevention, prompt diagnosis and detection of drug resistance, and superior treatment of TB. These various factors converged to produce the unprecedented resurgence of TB experienced in the United States during 1985–1992 (*33*). The rapid dissemination of multidrug-resistant TB among HIV-infected persons and their caregivers was accompanied by unacceptably high mortality rates and served as a clarion call to elicit concerted efforts and mobilize new resources to implement the 1992 National Action Plan to Combat Multidrug Resistant TB (*34*). The US Federal TB task force coordinated interagency work and successfully worked with health department-based TB programs across the nation to reverse this trend over ensuing years. In 2000, the Institute of Medicine reaffirmed the goal of TB elimination and recommended additional steps required for accelerated progress, including the need to commit to elimination as a national goal and to monitor progress (*35*).

In recent years, the risk of renewed complacency, resource limitations experienced by local health departments, and the direct effects of global TB on US disease rates (nearly 60% of incident TB cases reported in the United States in 2009 occurred in foreign-born persons) challenges advances to TB elimination in the near future. Bold ambition and expectations with sustained actions are a requisite to successfully eliminating TB in the United States and globally. The report of the 1997 Dahlem Workshop on the Eradication of Infectious Diseases recognizes that “[t]he success of any disease eradication initiative depends strongly on the level of societal and political commitment… Elimination and eradication are the ultimate goals of public health, evolving naturally from disease control. The basic question is whether these goals are to be achieved in the present or some future generation” (*36*).

Smallpox is the only infectious disease in humans that has been successfully eradicated, and this was only achieved by a campaign characterized by global solidarity in planning, collaboration, and concerted action. Few other infectious diseases meet the conditions that favor elimination or eradication (*36*). For the first time in history, the international community has developed an impressive plan to eliminate global TB (*14*). We must seize this opportunity to make added and continued progress against this global health scourge. A nonconformist stance must prevail until TB is eliminated. This frame of mind was aptly recognized in 1963 by William Brown, who advocated for syphilis eradication during the 1960s. He argued that diseases targeted for eradication (or elimination) should attain a “status of intolerability” by both health authorities and the public, such that any occurrence of the disease, “no matter how small,” gives cause for immediate action (*37*). Public clamor would help ensure sustained political commitment and ongoing work. With relatively few exceptions, this sense of bold ambition has not characterized those working in TB prevention and control programs. A sense of impoverished will tends to afflict those who work in resource-limited settings. And, to add insult to injury, there is a natural human propensity toward complacency when progress is being made and a disease is perceived to be under control. Attention and resources risk being diverted to address other pressing health needs. A 1962 Time Magazine article on syphilis resurgence and prospects for eradication demonstrates Dr Brown’s full grasp of this reality when he stated: “As a program for the control of a disease approaches the end point, meaning eradication, it is not the disease but the program that is the more likely to be eradicated” (*38*).

Clearly, we must boldly aspire to achieve the elimination of TB and commit to making it a reality in the United States and throughout the globe. Bridging all 3 gaps in implementation, knowledge, and ambition should become mutually reinforcing to achieve the desired results.
